# Alternate routes of influenza A virus infection in Mallard (*Anas platyrhynchos*)

**DOI:** 10.1186/s13567-018-0604-0

**Published:** 2018-10-29

**Authors:** Michelle Wille, Caroline Bröjer, Åke Lundkvist, Josef D. Järhult

**Affiliations:** 1 Zoonosis Science Center, Department of Medical Biochemistry and Microbiology, Uppsala University, Uppsala, Sweden; 2 Department of Pathology and Wildlife Diseases, National Veterinary Institute (SVA), Uppsala, Sweden; 3 Section for Infectious Diseases, Department of Medical Sciences, Uppsala University, Uppsala, Sweden; 4 Present Address: WHO Collaborating Centre for Reference and Research on Influenza, At the Peter Doherty Institute for Infection and Immunity, Melbourne, Australia

## Abstract

The natural reservoir for all influenza A viruses (IAVs) is wild birds, particularly dabbling ducks. During the autumn, viral prevalence can be very high in dabbling ducks (> 30%) in the Northern Hemisphere, and individuals may be repeatedly infected. Transmission and infection is through the fecal–oral route, whereby birds shed viruses in feces and conspecifics are infected though feeding in virus-contaminated water. In this study we wanted to assess two alternative infection routes: cloacal drinking and preening. Using experimental infections, we assessed patterns of infection using a combination of virus shedding, as assessed by real-time PCR from cloacal swabs, and patterns of viral replication using virus-immunohistochemistry of gastrointestinal tissues. The cloacal drinking experiment consisted of two trials using cloacal inoculation at two different time points to account for age differences, as well as a trial whereby ducks were allowed to take up virus-laden water through the cloaca. All ducks became infected, and rather than the bursa of Fabricius being the main site of replication, the colon had the highest intensity of replication, as inferred through immunohistochemistry. In experiments assessing preening, feathers were contaminated with virus-laden water and all ducks became infected, regardless of whether they were kept individually or together. Further, naive contacts were infected by the individuals whose feathers were virus-contaminated. Overall, we reinforce that IAV transmission in dabbling ducks is multifactorial—if exposed to virus-contaminated water ducks may be infected through dabbling, preening of infected feathers, and cloacal drinking.

**Electronic supplementary material:**

The online version of this article (10.1186/s13567-018-0604-0) contains supplementary material, which is available to authorized users.

## Introduction

Influenza A virus (IAV) is one of the most important viruses of the twentieth century [1]. It is most conspicuous in food production animals, such as poultry, due to high morbidity and mortality and subsequent socioeconomic losses [2–5], but the natural reservoir of all IAVs are wild birds, specifically dabbling ducks [6, 7]. Wild birds are infected with low pathogenic IAV, with no clinical signs of disease [6, 8], in contrast to highly pathogenic IAV which is maintained in, and associated with high mortality in poultry. Low pathogenic IAVs replicate in the gastrointestinal tract [7, 9, 10], specifically, the intestinal epithelium from the ileum to colon, and the surface epithelium of the bursa of Fabricius of young birds, without causing gross or microscopic lesions [11]. Furthermore, low pathogenic IAVs in wild birds do not cause respiratory infections [9, 10, 12]. Infection is acute and is usually cleared within 7 days [7, 13]. Overall, it is hypothesized that there has been a long co-evolution between waterfowl and IAV [14], resulting in a great subtype and lineage diversity and low virulence of these viruses in the waterfowl reservoir.

The success of avian IAV is, in part, due to efficient transmission of virus between waterfowl hosts. In dabbling ducks, transmission is thought to be largely water-borne and to occur through the fecal–oral route [7, 10, 15]. That is, virus is shed through the feces into the water [10], and while dabbling, ducks concentrate virus-contaminated water and subsequently ingest virus [7, 16]. Dabbling ducks in particular utilize fresh or brackish water, which may allow better survival of IAV [7, 10]. Furthermore, the shape of the bill of dabbling ducks, which contain lamellae, may be important in concentrating viruses. Indeed, species with a higher laminar density have been correlated with higher IAV prevalence [16]. Given the aquatic nature of waterfowl, specifically the utilization of potentially virus contaminated water bodies, two alternative routes of transmission have been proposed: cloacal drinking and preening. First, Daoust et al. [11] proposed that cloacal drinking, or the uptake of fluids through the cloaca, may be an important route for infection of the bursa of Fabricius. It has been noted that in young birds infection may be localized to the bursa of Fabricius, with no evidence of infection in the gastrointestinal tract [11, 12]. The bursa of Fabricius is important in B cell development, and atrophies prior to maturity [17–19]. While cloacal drinking has not been assessed in ducks in the context of IAV, cloacal drinking has been shown in chickens [20], and may be important in the transmission of protozoan *Histomonas meleagridis* causing Blackhead in poultry [21].

Second, Delogu et al. [22] hypothesized that preening behavior could facilitate IAV accumulation on duck feathers. Specifically, they showed that attachment of IAV to feathers from contaminated water is facilitated by preening oil produced by the uropygial gland. Indeed, both low pathogenic and highly pathogenic IAV have successfully been isolated from feathers in wild and experimental settings [23–26]. Lebarbenchon et al. [23] further proposed this could be an alternative method for detecting viruses in wild duck populations. Nuradji et al. [26] found that viral titres of highly pathogenic IAV were higher in feather samples than in swabs, however, the proportion of positive feathers was lower than the prevalence in oropharyngeal or cloacal swabs. Interestingly, the importance of feathers has been incorporated into policy, whereby some countries restrict the import of feathers specifically due to IAV infection risk [27, 28].

**Figure 1:**
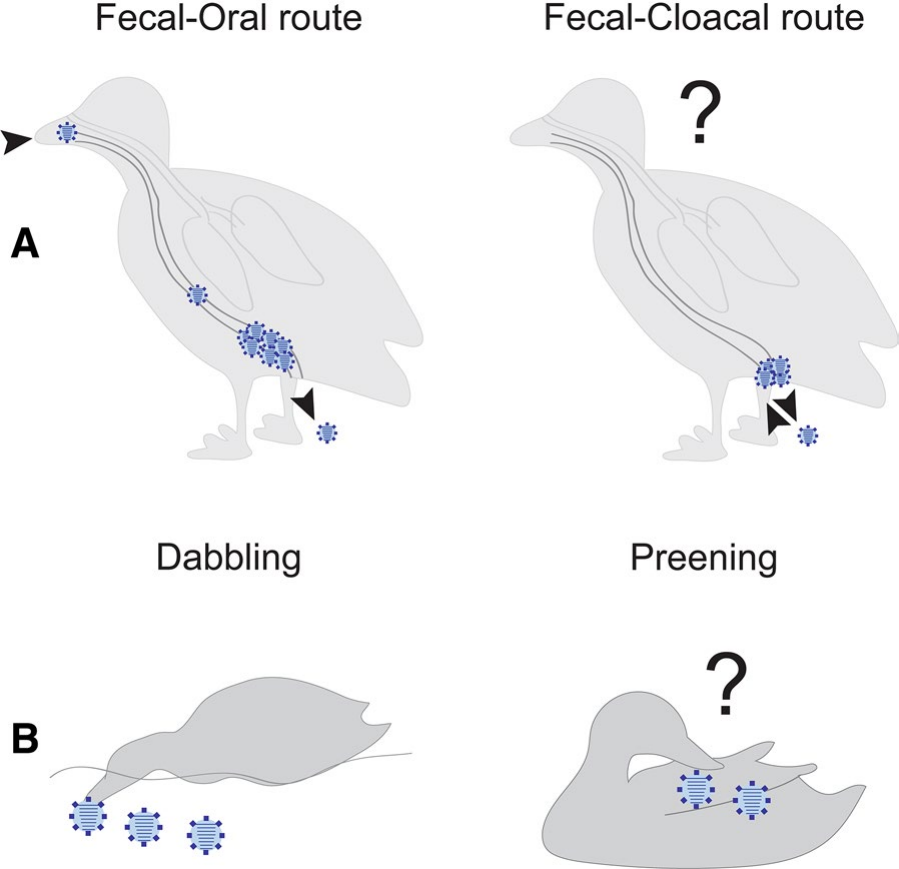
**Conceptual questions raised in this study. A** Whether birds may be infected directly through the cloaca, expanding the accepted fecal–oral route of transmission, and **B**, given the fecal–oral route of transmission, whether birds may become infected through preening, rather than being limited to water-bourne transmission and dabbling.

In this study we aimed to assess alternative routes of transmission of IAV using a Mallard (*Anas platyrhynchos*) model as this species is the central dabbling duck reservoir [6] (Figure 1). Specifically, we aimed to broaden the transmission concept beyond the classic fecal–oral route and assess whether (1) cloacal drinking may play a role and explain why some young birds are only infected in the bursa of Fabricius, and (2) birds may be infected through preening. As such, we demonstrate that the promiscuous nature of IAV transmission and mode of infection in the natural reservoir is consistent with a successful and long-evolved host–pathogen interaction.

## Materials and methods

### Experimental conditions

Wild-strain domestic Mallard ducklings were purchased from a domestic (Swedish) breeder, and were raised at the National Veterinary Institute (SVA), Uppsala, Sweden. Prior to the start of experiments, all individuals tested negative for IAV antibodies by NP-ELISA (IDEXX, Avian Influenza Virus Antibody Test Kit, Hoofddorp, The Netherlands). At the start of the experiment, ducks were moved to HEPA filtered rooms with negative air pressure. Mallards were housed indoors at a BSL2 animal facility at SVA with a 12 h day–night cycle and had access to food ad libitum. No swimming pool was provided but drinking water was provided as required by the Swedish Board of Agriculture. In order to limit IAV contamination of drinking water, drinkers were decontaminated and fresh water was provided daily. Drinkers were additionally raised to prevent defecation into drinking water and prevent ducks from bathing in the water, i.e., prevention of the fecal–oral route.

Cages used in some of the experiments were modified rabbit cages, and conformed to the minimum size requirements per individual, as outlined by the ethical approval and regulations by the Swedish Board of Agriculture. The floor of the cages was perforated with holes so that any spilled water or feces would fall through the cage floor to limit accidental transmission by the fecal–oral route. Each Mallard had a mirror, and all individuals could see at least one other individual across the room and hear the ducks inside the room. There were five duck cages in the experimental room. To assess transmission between conspecifics, Mallards in the preening transmission trial were placed into an experimental room, where they were able to move around freely and interact with their conspecifics. All cages and rooms were cleaned after 24 h. At 48 h, ducks in all experiments were euthanized with an overdose of pentobarbital (Allfatal vet, Omnidea AB).

### Virus preparation

Virus stock used in all experiments was 10^8^ EID_50_/mL of A/Mallard/Sweden/101663/2009 (H4N6). This virus was isolated from wild migratory Mallards at Ottenby Bird Observatory in Sweden; 200 μL of the original sample in virus transport media (VTM) was inoculated in embryonated chicken eggs via the allantoic route. The allantoic fluid was harvested after 2 days, and confirmed by agglutination. Virus stock for duck experiments was grown up from the original isolate (thus second passage; E2) and 50% Embryo Infectious Dose (EID_50_) was calculated according to Reed and Muench [29].

### Experimental design: cloacal drinking

In order to assess our first hypothesis, that cloacal drinking is a viable transmission route, and to assess the infection of cloacal bursas of young ducks, a two-part experiment was performed (Figure 1A). First, Mallards were infected using cloacal inoculation. To account for putatively different sizes of the bursa of Fabricius, ducks were divided into two age categories, 4 (*n* = 5) and 6 months (*n* = 5). Cloacal inoculations were performed by slowly injecting 2 mL of virus stock into the cloaca with a blunt-end metal canula (normally used for oesophageal inoculation [30]). Following inoculation, we waited 1 min to allow ducks to eject any superfluous material from the cloaca, and then the feathers around the cloaca were sprayed and cleaned with ethanol. In the second part of the experiment we attempted to mimic the natural route of cloacal drinking (henceforth cloacal exposure) using ducks aged 5 months (*n* = 4). Each individual was placed in a custom-made enclosed box with a flexible collar, preventing the duck from creating water drops through splashing and isolating the head from the virus-laden water (Additional file 1). Boxes were filled with virus laden water (2 L, 3 × 10^5^ EID_50_) (Table 1). Mallards were “inoculated” in the boxes for 1 h, with the lights off to reduce stress. Personnel were in the room during exposure in order to terminate the experiment in case signs of severe distress were observed. Following the 1-h exposure period, each duck was removed from the box, all the excess water dried off, and the feathers sprayed thoroughly with ethanol to remove any viral residue. If it was suspected that an individual may have been infected oesophageally it was removed from the experiment and was excluded from subsequent rRT-PCR or immunohistochemistry (IHC) analysis to remove bias. Each duck was subsequently placed into an individual cage for 48 h.

**Table 1:** **Experimental design for trials to assess alternate infection routes**

Experiment	Trial	# Ducks	Age of ducks (months)	Mode of inoculation	Housing
Cloacal drinking	Cloacal inoculation	5	4	Inoculation of 2 mL virus into the cloaca with canula	Each duck in a separate cage immediately following inoculation
	Cloacal inoculation	5	6	Inoculation of 2 mL virus into the cloaca with canula	Each duck in a separate cage immediately following inoculation
	Cloacal exposure	4	5	Placed in virus laden water (6 mL virus stock + 2 L water) in specially designed inoculation box	Each duck in a separate cage immediately following inoculation
Preening	Preening cages	5	6	Bathed with virus laden water (3 mL virus stock + 1 L water)	Each duck in a separate cage immediately following inoculation
	Preening transmission (inoculation)	5	6	Bathed with virus laden water (3 mL virus stock + 1 L water)	Ducks placed in an experimental room with conspecifics
	Preening transmission (contacts)	5	6	Unexposed contacts	Ducks placed in an experimental room with conspecifics

### Experimental design: preening

In order to assess the role of preening in infection (Figure 1B), ten 6 month old Mallards were bathed with virus-laden water (1 L, 3 × 10^5^ EID_50_), ensuring all feathers were visibly wet. While contaminating the feathers, care was taken to ensure that no virus-laden water entered the cloaca, eyes, nares or mouths of ducks, and to limit droplet formation. Following contamination of the feathers, Mallards were either (1) placed in individual cages (*n* = 5), (2) placed in a room where they could move around freely (*n* = 5) and (3) placed with ducks that were uninfected and not inoculated to act as contacts (*n* = 5) (Table 1).

### Detection and quantification of viral shedding

Ducks were sampled on three occasions: immediately prior to the start of the experiment [0 days post-infection (dpi)], at 24 h (1 dpi), and at 48 h (2 dpi). Cloacal samples were collected with a sterile tipped applicator and placed in virus transport media (Hank’s balanced salt solution containing 0.5% lactalbumin, 10% glycerol, 200 U/mL penicillin, 200 mg/mL streptomycin, 100 U/mL polymyxin B sulfate, 250 mg/mL gentamicin, and 50 U/mL nystatin; Sigma). Samples were stored at −80 °C within 4 h of collection. RNA was extracted using the MagNA Pure 96™ Nucleic Acid Purification System (Roche, Mannheim, Germany) and MagNA Pure 96 DNA and Viral Nucleic Acid Large Volume Kit (Roche) following manufacturer’s recommendations. Following extraction, samples were assayed by real time reverse transcriptase PCR (rRT-PCR) with the One Step RT-PCR Kit (Qiagen, Hilden, Germany), targeting a short fragment of the IAV matrix gene [31] on a Roche Light Cycler 480. A cycle threshold (Cq) value of less than 40 was considered positive.

### Histology and immunohistochemistry

Necropsies were performed immediately following euthanasia. From each individual we collected gastrointestinal tract tissues: two seven-cm-long segments of jejunum (J1 and J2) and two seven-cm-long segments of ileum (I3 and I4) at intervals of approximately seven centimeters apart, colon (C), and cloacal bursa (B). All tissues were fixed in 10% neutral-buffered formalin (Sigma) for histopathology and IHC. Briefly, after fixation, samples were processed routinely, embedded in paraffin, and sectioned at 4–5 µm. Sections were processed for IHC using a commercial anti-influenza A nucleoprotein primary monoclonal antibody (HB65, EVL, Woerden, The Netherlands). Detailed histopathology and IHC methods are described in Bröjer et al. [32].

Cells with distinct red staining in the nucleus or cytoplasm were identified as sites of virus replication, and tissues were considered positive even if only one or a few positive cells were present. The intensity and extension of the immunostaining was assessed semiquantitatively by the following scoring system: 0: no positive cells, 0.5: positive cells present, 1: mild, 2: moderate, 3: marked.

### Statistics

Variation in Cq values across dpi within each trial were tested using a paired t-test and across trials within dpi were tested using ANOVA, following a test to ensure normal distribution of residuals. Two-way ANOVAs were not used due to small sample sizes. *p* values of < 0.05 were taken to indicate a significant difference in the compared rates. Statistics were done using GraphPad Prism v.6.03 (GraphPad Software Inc, San Diego, CA, USA).

## Results

### IAV shedding

**Figure 2:**
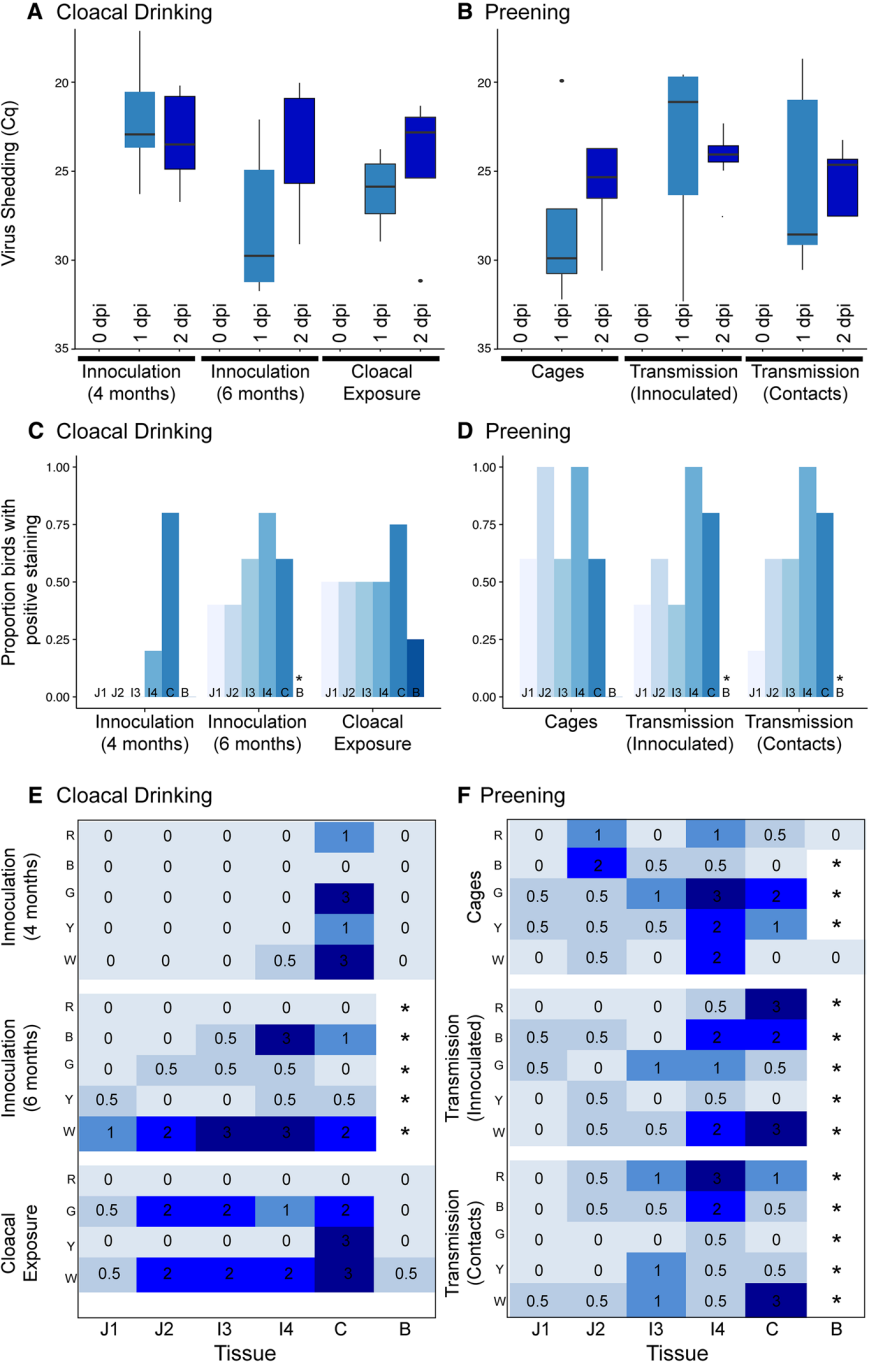
**Patterns of infection in experimental Mallards. A**, **C**, **E** Refer to cloacal drinking experiments, **B**, **D**, **F** refer to the preening experiments. **A**, **B** Virus shedding, represented by Cq values on an inverted Y-axis, where a low Cq value is indicative of high levels of shedding. For each trial, box and whiskers plots are shown for each day of the experiment which illustrate the median (black line) and spread of the data (upper and lower quartiles). Different colours correspond to different days. **C**, **D** Proportion of individuals with positive staining for IAV in each tissue across the gastrointestinal tract. The X axis is divided into experimental trial, and within trials subdivided into tissue from different parts of the gastrointestinal tract, J1 to B, ranging from light blue to dark blue. **E**, **F** Heatmap of intensity of infection as inferred by relative number of positive cells for each individual and tissue. Values range from 0 (no positive staining), 0.5 (positive cells present), 1 (mild), 2 (moderate), to 3 (marked staining). Each individual for each experiment is plotted along the Y axis (R, B, G, Y, W), and tissue type across the X axis. For **C**–**F**, tissues assessed are two sections of jejunum J1, J2, two sections of ileum I3, I4, colon C, and bursa B. An asterisk indicates no bursal tissue could be identified. In the final cloacal drinking trial, only four ducks were included, where all other experiments have *n* = 5. Intensity scores and Cq values for individual ducks are presented in Additional file 2.

Cloacal swabs collected from all birds were negative by rRT-PCR at 0 dpi, directly prior to exposure to IAV. Cloacal swabs from all birds, regardless of experiment or treatment, were positive for IAV at 1 dpi and 2 dpi (Figures 2A and B). Patterns of shedding were not significantly different for different treatment groups or days, for either the preening or cloacal drinking experiments, potentially due to few replicates and, for some groups, a large spread in the data. Specifically, there was no statistical difference between 1 and 2 dpi for the cloacal drinking trials (4 months t = 0.6689, *p* = 0.5402; 6 months t = 1.3218, *p* = 0.2568; exposure t = 0.5021, *p* = 0.6501) or the preening trials (cages t = 1.2765, *p* = 0.2708; innoc t = 0.0303, *p* = 0.9773; contact t = 0.0643, *p* = 0.9518). Despite apparent, but not significant variation at 1 dpi (Cloacal drinking 1 dpi *F*_*2,11*_= 3.607, *p* = 0.0624 and Preening 1 dpi *F*_*2,12*_= 1.397, *p* = 0.2848), all treatments had a similar range of Cq values at 2 dpi (Cloacal drinking 2 dpi *F*_*2,11*_= 0.1708, *p* = 0.8452 and Preening 2 dpi *F*_*2,12*_= 1.387, *p* = 0.2871) (Figures 2A and B).

### Patterns of replication

As a complement to viral shedding (Cq values), we assessed sections of the gastrointestinal tract using IHC for localization and intensity of infection. Patterns of viral replication suggest that cloacal inoculation does initiate infection, given that a large proportion of both 4 and 6 month old ducks had discernible positive cells (Figure 2C) and all ducks shed virus (Figure 2A, Additional file 2). In the cloacal exposure trial we allowed ducks to take up virus-laden water rather than directly inoculating virus into the cloaca. Viral replication showed two trends (Figure 2E). The first was no positive cells (Mallard R) or positive cells restricted to the colon (Mallard Y), which was more similar to patterns of replication in the cloacal inoculation trials. The second pattern (Mallard G and Mallard W) had marked positive staining throughout the gastrointestinal tract, and this pattern was more similar to birds in the preening experiment (Figure 2F). One individual in each of the drinking trials had no staining in any tissues (Cloacal inoculation 4 months Mallard B, Cloacal inoculation 6 months Mallard R, Cloacal exposure Mallard R). These individuals did shed virus (as detected by rRT-PCR), but had higher Cq values than other individuals in the same trial (Additional file 2).

Overall, when present (birds 4 and 5 months old) the bursa of Fabricius was free of IAV replication, except for scant replication observed in one individual. While bursal tissue could be identified in ducks at 4 and 5 months, 18/20 ducks that were 6 months (cloacal inoculation and preening trials) had no discernible bursal tissue (Figures 2E and F and Additional file 2), suggesting that the bursa had atrophied. There appeared to be more widespread replication in these older ducks (6 months old), with scant positive staining found in both the small intestine and colon.

**Figure 3:**
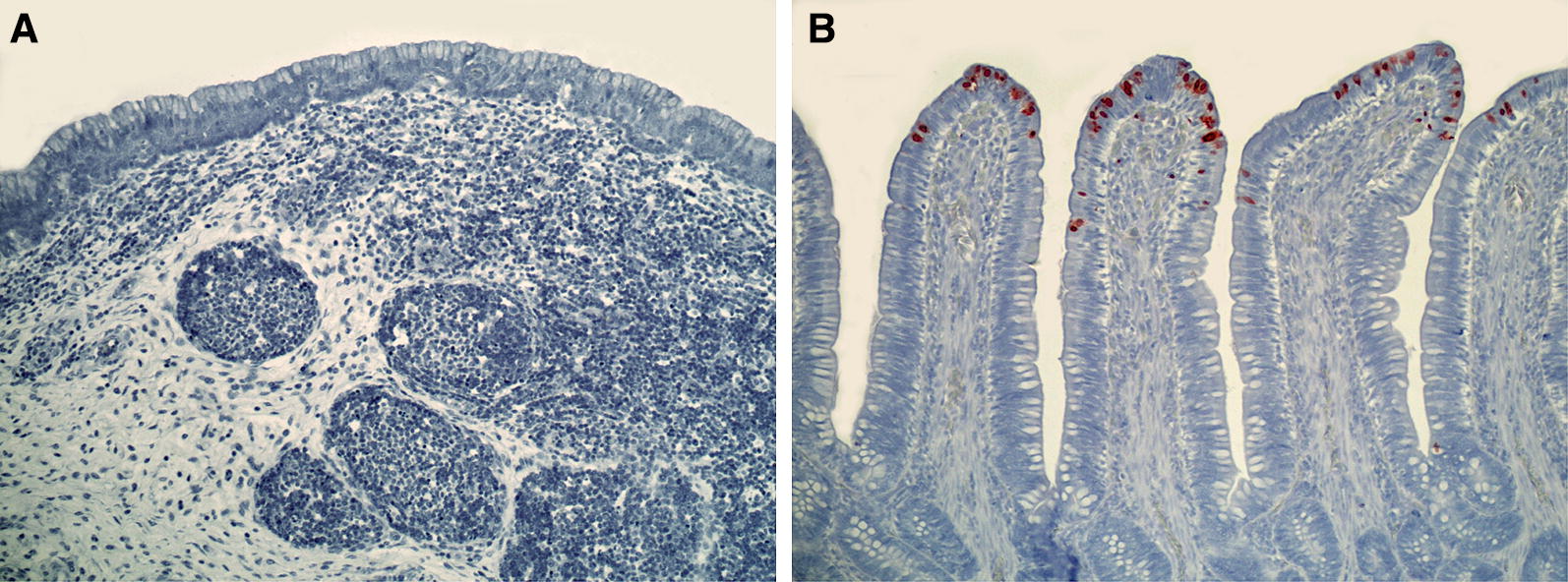
**Select tissues following immunohistochemical staining to detect nucleoprotein of influenza A. A** The bursa of Fabricius, when present, did not show virus antigen expression. **B** Positive staining of surface epithelium on the tips of villi in the colon. This tissue was scored as a “3” as positive staining is “marked”. Viral antigen expression is inferred by red staining in the nucleus of surface epithelium. Tissues have been counterstained with hematoxylin and appear blue. Original magnification is 200×.

In the preening experiments, regardless of treatment (cages, inoculated transmission group or negative contact birds in the transmission group), positive staining was found throughout the gastrointestinal tracts of all individuals (Figure 2E), and for each tissue, in a large proportion of individuals (Figure 2D). The largest number of positive cells was found in the ileum anterior to the caecal junction (I4) and colon (Figures 2D and F). In all experiments, positive cells were located in the surface epithelium, particularly on the tips of the villi, and in some section of the small intestine, also in the lamina propria (Figure 3).

## Discussion

One reason for the success of IAV in the waterfowl reservoir is its transmission efficiency, potentially driven in part by flexibility in mode of transmission and infection. In nature, water provides an important conduit, whereby ducks are infected through virus-contaminated water following dabbling. Indeed, uninfected ducks quickly become infected if sharing water with infected conspecifics, a premise which surveillance of sentinel ducks relies upon [33–35]. The fecal–oral route, linked closely with dabbling, is central to transmission and infection. We illustrate that transmission route and subsequent infection is not restricted to the fecal–oral route and dabbling by demonstrating infection following cloacal drinking and preening in this study.

Cloacal drinking has been shown to be an infection route in birds for other diseases such as blackhead [21], and for IAV it has been hypothesized to be important in young birds wherein infection may be localized to the bursa of Fabricius [11, 12]. The bursa of Fabricius plays an important role in the immune system and is responsible for B cell formation. Unlike infectious bursal disease [36] or Marek’s Disease [37] which target the bursa due to an abundance of immune cells such as B-lymphocytes, IAV infects the same cells in the bursa as in the gastrointestinal tract, i.e., the surface epithelium [11]. In chickens, the bursa of Fabricius atrophies prior to maturity, at about 6 months [18, 19], and in this study we were unable to locate the bursa of Fabricius in 18/20 Mallards that were about 6 months old. We did, however identify this organ in Mallards at 4 and 5 months of age. Interestingly, in individuals in which the bursa of Fabricius was identified, none or only scant numbers of cells were found to be positive in IHC-stained sections, despite inoculation directly into the cloaca. Colon seemed to be the most important site of replication even if all other tissues were negative. Further, in the cloacal exposure trials, whereby individuals took up water through their cloaca, the birds did not have staining in the bursa of Fabricius, except a single individual which had similar staining in the colon only. Our results, therefore, do not support the hypothesis whereby localized infection in the bursa of Fabricius is due to cloacal drinking. An alternative hypothesis is that following intense infection in the gastrointestinal tract, the final site of viral replication may be the bursa [12]. However, our results support that dabbling ducks can be infected through cloacal inoculation as well as cloacal drinking.

The results from the cloacal exposure trial were harder to interpret given that two of the individuals had very intense replication along the entire length of the gastrointestinal tract, markedly different from the cloacal inoculation trials. A possible explanation is that these birds became infected following preening or through droplets; that is, we were unable to remove all virus from feathers, feather oils in particular may have prevented out ability to remove all the virus effectively, or during the process of removal from inoculation boxes ducks created aerosols and were thus oesophagally infected. Alternatively, it could be due to individual differences in infection patterns or in susceptibility in the Mallards despite all individuals being from the same breeder [35]. Given the potential for accidental oesophageal infection, alternative ways to test this are improvements in the cloacal drinking trials eliminating the possibility of preening behavior, or manipulating ducks to entirely prevent infection from preening or aerosols. However, the combined inoculation and drinking trials did illustrate that ducks are able to take up virus-laden water through cloacal drinking, and it is thus an alternate infection route.

As hypothesized, following contamination of feathers with virus-laden water all ducks in all treatment groups were rapidly infected, with widespread IHC staining along the gastrointestinal tract. Birds in separate cages had similar patterns of infection to birds cohoused, and acted as individual replicates thus better eliminating the possibility of a contaminated drinking water source. All cohoused birds in the preening trial, including both the infected birds and the uninfected conspecifics had similar patterns, both in viral shedding and IHC patterns of infection. Patterns of infection in this experiment were very similar to those described in intra-oesophageal infection [38]. The similarities were initially surprising as ducks in this study were not provided with a swimming pool and drinking water was raised in an attempt to limit contamination in order to prevent infection by drinking or dabbling virus laden water. One hypothesis for the similarity is that once infected, ducks have similar virus infection curves. Therefore, regardless of whether dabbling or preening, individuals concentrate virus in their bill lamella [16] and then ingest virus. Delogu et al. [22] propose that viruses adsorb to bird feathers and are further concentrated on the feathers by AIV “sticking” to uropygial gland secreted oils that are used to protect and waterproof feathers. As such both concentrating virus on feathers, and subsequently in bill lamella may work synergistically. One study found that Mallards spend 10.9% of their day preening, including partaking in allopreening (that is, preening of other individuals) [39], and thus this may be an important supplementary infection route in dabbling ducks such as the Mallard. Not all waterfowl feed by dabbling, such as swans, geese or diving ducks, and preening may be a significantly more important transmission route for these waterfowl [22].

We demonstrate that IAV transmission in dabbling ducks is multifactorial. If exposed to virus-contaminated water, ducks may be infected through dabbling, preening of infected feathers, and cloacal drinking. Furthermore, even without a virus contaminated water source, contact ducks were infected by their conspecifics illustrating the role of direct contact, preening and allopreening. The findings are important to consider when conducting and interpreting surveillance and transmission studies of IAV in the natural host. Surveillance using feathers may, for example, be a useful tool in describing the viral diversity. This study highlights the ubiquitous nature and flexibility of IAV transmission in the Mallard host, consistent with a long history of host–pathogen co-evolution.

## Additional files

**Additional file 1.** **Schematic design for the purpose-built inoculation boxes for the cloacal drinking experiment.**

**Additional file 2.** **Intensity of infection for all individuals in the study.**
