# Supplementary figures and images for: Alternate routes of influenza A virus infection in Mallard (Anas platyrhynchos)
Source: Vet Res. 2018 Oct 29;49:110. doi: 10.1186/s13567-018-0604-0 (PMC6206871; doi:10.1186/s13567-018-0604-0)

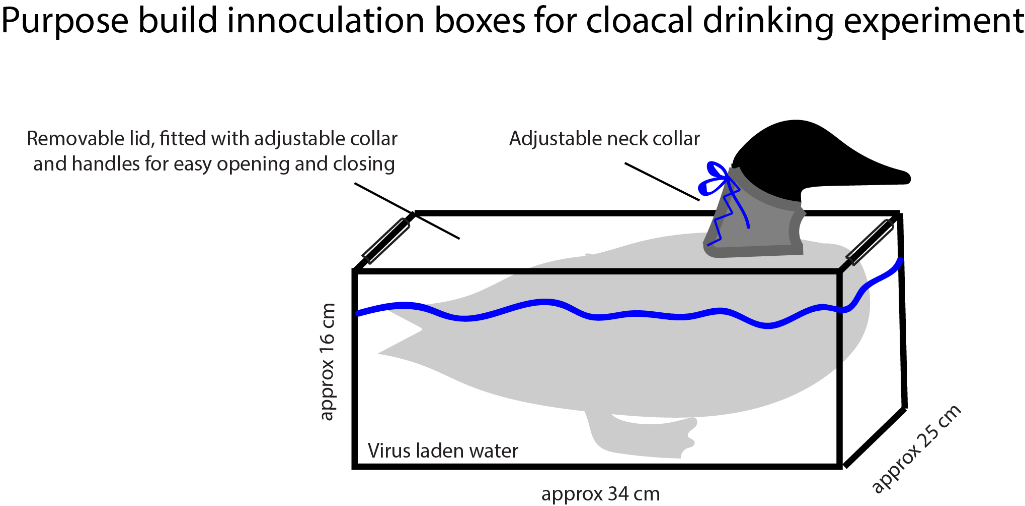

Supplement: Supplementary file 1 — Additional file 1. Schematic design for the purpose-built inoculation boxes for the cloacal drinking experiment. [file 13567_2018_604_MOESM1_ESM.docx]
