# Supplementary material for: Alternate routes of influenza A virus infection in Mallard (Anas platyrhynchos)
Source: Vet Res. 2018 Oct 29;49:110. doi: 10.1186/s13567-018-0604-0 (PMC6206871; doi:10.1186/s13567-018-0604-0)
Supplement: Supplementary file 2 — Additional file 2. Intensity of infection for all individuals in the study. [file 13567_2018_604_MOESM2_ESM.docx]

| Trial | Individual | Duck Age | Ct @ 2dpi | Intensity score based on positive staining^1^ | | | | | |
| --- | --- | --- | --- | --- | --- | --- | --- | --- | --- |
|  |  | (months) |  | J1^2^ | J2^2^ | I3^2^ | I4^2^ | Colon | Bursa |
| Cloacal Inoculation (4 months) | R | 4 | 20,19 | 0 | 0 | 0 | 0 | 1 | 0 |
| Cloacal Inoculation (4 months) | B | 4 | 26,72 | 0 | 0 | 0 | 0 | 0 | 0 |
| Cloacal Inoculation (4 months) | G | 4 | 23,5 | 0 | 0 | 0 | 0 | 3 | 0 |
| Cloacal Inoculation (4 months) | Y | 4 | 24,88 | 0 | 0 | 0 | 0 | 1 | 0 |
| Cloacal Inoculation (4 months) | W | 4 | 20,8 | 0 | 0 | 0 | 0.5 | 3 | 0 |
| Cloacal Inoculation (6 months) | R | 6 | 29,1 | 0 | 0 | 0 | 0 | 0 | No bursa |
| Cloacal Inoculation (6 months) | B | 6 | 20,91 | 0 | 0 | 0.5 | 3 | 1 | No bursa |
| Cloacal Inoculation (6 months) | G | 6 | 25,68 | 0 | 0.5 | 0.5 | 0.5 | 0 | No bursa |
| Cloacal Inoculation (6 months) | Y | 6 | 25,68 | 0.5 | 0 | 0 | 0.5 | 0.5 | No bursa |
| Cloacal Inoculation (6 months) | W | 6 | 20,04 | 1 | 2 | 3 | 3 | 2 | No bursa |
| Cloacal Exposure | R | 5 | 31,16 | 0 | 0 | 0 | 0 | 0 | 0 |
| Cloacal Exposure | G | 5 | 21,22 | 0.5 | 2 | 2 | 1 | 2 | 0 |
| Cloacal Exposure | Y | 5 | 23,46 | 0 | 0 | 0 | 0 | 3 | 0 |
| Cloacal Exposure | W | 5 | 22,18 | 0.5 | 2 | 2 | 2 | 3 | 0.5 |
| Preening Individual | R | 6 | 30,59 | 0 | 1 | 0 | 1 | 0.5 | 0 |
| Preening Individual | B | 6 | 23,73 | 0 | 2 | 0.5 | 0.5 | 0 | No bursa |
| Preening Individual | G | 6 | 25,34 | 0.5 | 0.5 | 1 | 3 | 2 | No bursa |
| Preening Individual | Y | 6 | 26,52 | 0.5 | 0.5 | 0.5 | 2 | 1 | No bursa |
| Preening Individual | W | 6 | 23,71 | 0 | 0.5 | 0 | 2 | 0 | 0 |
| Preening Transmission (inoculated) | R | 6 | 24,06 | 0 | 0 | 0 | 0.5 | 3 | No bursa |
| Preening Transmission (inoculated) | B | 6 | 24,96 | 0.5 | 0.5 | 0 | 2 | 2 | No bursa |
| Preening Transmission (inoculated) | G | 6 | 23,57 | 0.5 | 0 | 1 | 1 | 0.5 | No bursa |
| Preening Transmission (inoculated) | Y | 6 | 24,48 | 0 | 0.5 | 0 | 0.5 | 0 | No bursa |
| Preening Transmission (inoculated) | W | 6 | 22,32 | 0 | 0.5 | 0.5 | 2 | 3 | No bursa |
| Preening Transmission (contacts) | R | 6 | 24,33 | 0 | 0.5 | 1 | 3 | 1 | No bursa |
| Preening Transmission (contacts) | B | 6 | 30,75 | 0 | 0.5 | 0.5 | 2 | 0.5 | No bursa |
| Preening Transmission (contacts) | G | 6 | 26,02 | 0 | 0 | 0 | 0.5 | 0 | No bursa |
| Preening Transmission (contacts) | Y | 6 | 30,77 | 0 | 0 | 1 | 0.5 | 0.5 | No bursa |
| Preening Transmission (contacts) | W | 6 | 27,59 | 0.5 | 0.5 | 0 | 0.5 | 3 | No bursa |

^1^ Intensity scores were assigned as follows: 0 - no positive cells, 0.5 - positive cells present, 1 - mild, 2 - moderate, 3 - marked

^2^ Two seven-cm-long segments of jejunum (J1 and J2) and two seven-cm-long segments of ileum (I3 and I4) at intervals of approximately seven centimeters apart
